# Quality analysis and metabolomic profiling of the effects of exogenous abscisic acid on rabbiteye blueberry

**DOI:** 10.3389/fpls.2023.1224245

**Published:** 2023-07-10

**Authors:** Hao Yang, Tianyu Han, Yaqiong Wu, Lianfei Lyu, Wenlong Wu, Weilin Li

**Affiliations:** ^1^ Co-Innovation Center for Sustainable Forestry in Southern China, College of Forestry, Nanjing Forestry University, Nanjing, China; ^2^ Institute of Botany, Jiangsu Province and Chinese Academy of Sciences (Nanjing Botanical Garden Mem. Sun Yat-Sen), Jiangsu Key Laboratory for the Research and Utilization of Plant Resources, Nanjing, China

**Keywords:** ABA, anthocyanins, flavonoid, antioxidant capacity, blueberry

## Abstract

Blueberry is a characteristic berry fruit shrub of the genus *Vaccinium* in the Rhododendron family. The fruit is rich in anthocyanins and has a variety of nutritional and health functions. This study aimed to systematically study the effect of exogenous abscisic acid (ABA) application on ripening and metabolites in blueberry fruits. Blueberry fruit ripening was divided into six stages for further analysis. In this study, nontarget metabolomics was performed to demonstrate the effect on metabolite levels. The results showed that 1000 mg/L ABA significantly promoted fruit ripening and increased anthocyanin content. Moreover, exogenous ABA treatment can affect endogenous ABA levels and improve its antioxidant capacity. Important metabolites of the flavonoid pathway were detected, and the results showed that anthocyanin synthesis increased, and some other bioactive metabolite levels decreased. After comprehensive assessments, we believe that 1000 mg/L exogenous ABA application will have positive impacts on blueberry fruit quality and economic benefits.

## 1 Introduction

Blueberry is the common name of plants with purple- or blue-colored berries (Luby et al., 1991). Cultivated blueberries belong to the genus *Vaccinium* and mainly including lowbush blueberries, southern highbush blueberries, northern highbush blueberry plants, and rabbiteye blueberries (Leisner et al., 2017). In recent years, an increasing number of clinical and animal model studies have shown that blueberry fruit and its extracts have positive effects on the development of certain cancer (Wang et al., 2017) and obesity complications, such as chronic inflammation (Kang et al., 2015), type 2 diabetes (Shi et al., 2017) and cardiovascular disease (Thandapilly et al., 2022).

Blueberries are rich in dietary fibers, vitamins, minerals and bioactive compounds (including ascorbic acid, flavonoids, hydroxycinnamic acids, pterostilbene, resveratrol, and anthocyanins) (Nile and Park, 2014; Wu et al., 2022). Among them, anthocyanins play a major antioxidant role and are responsible for approximately 84% of the antioxidant capacity in blueberry (Borges et al., 2010). Blueberries have a high anthocyanin content and are rich in variety (up to 43 different anthocyanin components) (Neuenfeldt et al., 2022). The most common anthocyanidin aglycones are peonidins, pelargonidins, malvidins, delphinidins, cyanidins and petunidins (Yang et al., 2022). Purified blueberry anthocyanins alter the development of obesity in mice fed an obesogenic high-fat diet (Prior et al., 2010).

Plant fruits can be classically classified into two types according to the change patterns of respiration and ethylene during ripening: climacteric and non-climacteric fruits. In non-climacteric fruits, abscisic acid (ABA) plays a key role in fruit ripening (Zhang et al., 2021). Exogenous ABA was reported to affect the ripening of non-climacteric fruits in field experiments, such as grape (Wang et al., 2022), strawberry (Chen et al., 2022) and blackberry (Zhang et al., 2021). Influenced by exogenous ABA, the coloration, quality and anthocyanin content of non-climacteric fruits were changed (Yamamoto et al., 2015; Wang et al., 2022). As a kind of non-climacteric fruit, blueberry starts to accumulate anthocyanins from the ripening stage (Zifkin et al., 2012). Compared to research on exogenous ABA in grapes and strawberries, there are few studies on blueberries. Transcriptome sequencing and related transcription factor function studies clarified the mechanism by which exogenous ABA affects blueberry ripening and revealed that ABA can significantly induce the expression of anthocyanin related genes in fruits (Han et al., 2021). The fruits of southern highbush blueberry were treated with ABA at 200 ppm and 400 ppm during the green mature period, and the fruit number and the firmness of berries were found to be increased, suggesting a ripening delay effect (Buran et al., 2012). In northern highbush blueberry treated with 1000 mg/L ABA, coloration accelerated, and the anthocyanin content increased from the green mature period to the red period (12 days) (Oh et al., 2018).

Plant metabolites play essential roles in plants during growth, development and environmental stress (Wu et al., 2021). To adjust to different conditions, plants can change metabolites in response to environmental signals through hormone pathways (Ku et al., 2018). With the deepening understanding of the function, biosynthesis and metabolic mechanism of plant metabolites, metabolomics has been shown to be a powerful tool to understand how plants develop and respond to the environment at the metabolite level (Hong et al., 2016). Recently, metabolic level changes in chickpea (*Cicer arietinum*) under contrasting water regimes were demonstrated by nontargeted global ultrahigh-performance liquid chromatography/high-resolution mass spectrometry (UPLC–HRMS) (Khan et al., 2019). Liu et al. (2021) revealed the structural diversity and distribution of limonin in pomelo (*Citrus grandis*) fruit by comprehensive ultrahigh-performance liquid chromatography–tandem mass spectrometry (UHPLC−MS/MS) analysis. The effects of silver nanoparticles on cucumber (*Cucumis sativus*) were evaluated by gas chromatography−mass spectrometry (GC−MS)-based nontarget metabolomics (Zhang et al., 2018). Using LC−MS, it was found that the color difference in *Rubus* fruit was closely related to flavonoids (Wu et al., 2021). Wang et al. (2021), through widely targeted metabolomic analysis, revealed dynamic changes in nonvolatile and volatile metabolites during green tea processing.

Based on a previous study, the fruits of rabbiteye blueberry ‘Brightwell’ were treated with 0, 500, or 1000 mg/L exogenous ABA in the green mature period (Han et al., 2021). The main measured parameters included fruit skin color, fruit firmness, soluble solid content and total anthocyanin content. The 1000 mg/L ABA treatment was more significant than the 500 mg/L ABA treatment in the four parameters above (Han et al., 2021). Based on this, UHPLC−MS/MS was used to analyze the metabolite changes between 0 and 1000 mg/L exogenous ABA treatment in six stages in this study. By comparing metabolite changes, we thoroughly analyzed the effects of exogenous ABA on blueberry fruits from the green mature period to the purple mature period. This study provides new insight into the application of exogenous ABA to ripening non-climacteric fruits at the metabolite level.

## 2 Materials and methods

### 2.1 Plant materials and ABA treatments

**Figure 1:**
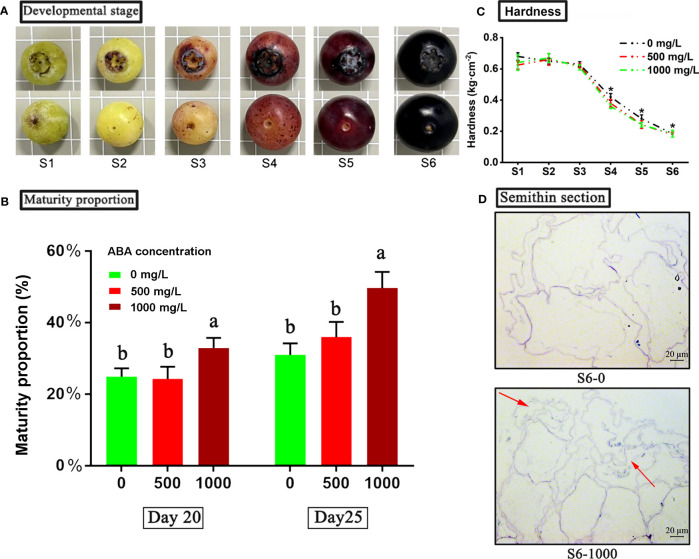
Physiological characterization of blueberry fruit in different treatment groups. **(A)** Six stages from late green fruit to full ripening of blueberry. An asterisk (*) indicates a significant difference between different treatments during the same period according to Tukey’s test (*P*< 0.05). **(B)** Effects of ABA treatments on the maturity ratio of blueberry fruits. Maturity ratio at 20 and 25 days after treatment. **(C)** The hardness of fruits under 0, 500 and 1000 mg/L ABA treatment from S1 to S6. **(D)** Semithin section observation of blueberry sarcocarp tissue at the S6 stage under ABA treatments. S6-0: control; S1000: 1000 mg/L ABA treatment. The red arrows indicate the areas of fragmentation and fragmentation of blueberry fruit flesh cells under 1000 mg/L ABA treatment compared to the control group.

In this study, the fruit of rabbiteye blueberry cultivar ‘Brightwell’ was used as the experimental material. Four-year-old blueberry trees in the Lishui District Baima Base of Institute of Botany, Jiangsu Province and Chinese Academy of Sciences were selected for sampling. The experiment was conducted in a completely randomized block design, with 6 replicates for each treatment and 6 plants for each block. At the late stage of green fruit development, the fruit was treated with 0 (control), 500 mg/L or 1000 mg/L ABA (95% purity, Coordinator Company, China) once, and the fruit was sprayed until the surface was wet. According to previous research results of this research group, blueberry fruits are divided into six stages from the late green fruit stage to the mature stage (Han et al., 2021). Specifically, in the first stage S1, the fruit turned green; in the second stage S2, the top of the fruit began to turn red, while the sides and bottom remained green. In the third stage S3, the side of the fruit begins to turn red; in the fourth stage S4, the fruit turns red as a whole. In the fifth stage S5, the entire fruit turns purplish red; in the sixth stage S6, the fruit turns completely dark purple (**Figure 1A**). At each fruit development stage, two groups of S1-S6 blueberry fruits treated with 0 or 1000 mg/L ABA were selected for six biological replications and stored at -75°C for further metabolomics experiments and analysis.

### 2.2 Physiological characterization

#### 2.2.1 Fruit hardness

The blueberry fruit hardness was measured by a fruit hardness tester (Cat no. 9300, Takemura Electric Works Ltd., Kyoto, Japan). A tapered tip was applied vertically to the side of the blueberry fruit (the probe diameter was 1 mm, and the pressing distance was 5 mm). The value was measured at the moment the tip penetrated the surface with the unit kg·cm^-2^.

#### 2.2.2 Fruit maturity rate

Based on the color and morphological changes during fruit maturity (S6 as the reference standard), the percentage of fully mature fruits in total fruits on the 20^th^ and 25^th^ days was calculated after ABA treatment. For each plant, one fruiting branch from each of the different orientations of the blueberry plant, east, west, south, north, top, middle and bottom, was selected to count the number of ripe fruits and calculate the fruit maturity proportion with the following formula:


Maturity proportion(%)=(Number of ripened fruits/Total number of fruits)×100%

#### 2.2.3 Light microscopy

Semithin section analysis was performed to observe the fruit pulp cells according to the method described by Pan et al. (2019). Briefly, 1 mm × 3 mm samples were fixed with 0.1 M phosphate buffer (pH 7.2) containing 2.5% (v/v) glutaraldehyde, washed three times with 0.1 M phosphate buffer and then soaked in 1% (v/v) osmic acid for 2 hours. Samples were washed again using 0.1 M phosphate buffer as described above and dehydrated through a gradient ethanol series. Ultimately, the samples were embedded and polymerized in Spurr’s resin. Semithin sections (2 μm) were stained with 1% methylene blue and then photographed under a Zeiss Axio Vert A1 microscope. Images are representative of six observed samples.

### 2.3 Reducing power assay

Reducing power assays were adapted from the method described by Shen et al. (2017), and ascorbic acid (0.1 mg/mL) was used as a positive control. Briefly, 0.15 g samples were homogenized with 1.35 mL of phosphate buffer (0.2 M, pH 6.6) and centrifuged at 5000 × g for 10 min. The supernatant (1 mL) was mixed with 1 mL of potassium ferricyanide solution (1%, w/v). The mixtures were then heated at 50°C for 20 min. Then, 1 mL of trichloroacetic acid (10%, w/v) solution was added to stop the reaction immediately. After centrifugation, 1 mL of supernatant was mixed with 1 mL of distilled water and 0.2 mL of ferric chloride solution (0.1%, w/v). After 10 min, the 96 plates were shaken sufficiently and measured spectrophotometrically at 700 nm.

### 2.4 DPPH radical scavenging activity assay

DPPH radical scavenging activity was measured according to Cerezo et al. (2010), and ascorbic acid (0.5 mg/mL) was used as a positive control. DDPH (0.1 mM) was dissolved in ethanol. The sample slurry was added to 3 DPPH reaction systems. Absorbance was measured at 515 nm after 30 min to reach a steady state.

The scavenging activity of the DPPH radical was calculated as follows:


$$\text{scavenging rate }\left(\%\right)=([1-\frac{Ai-Aj}{Ao}]\times 100)$$

where *Ai* is 1 mL ethanol + 1 mL DDPH solution, *Aj* is 1 mL sample slurry + 1 mL DDPH, and *Ao* is 1 mL sample slurry + 1 mL ethanol.

### 2.5 Metabolite extraction

Approximately 5 g of blueberry fruits from each treatment (a total of 72 fruit samples) was thoroughly ground with liquid nitrogen. Then, 100 mg of homogenate was collected and resuspended in prechilled 80% methanol and 0.1% formic acid (FA) by vortexing. The samples were incubated on ice for 5 min and then centrifuged at 15000 rpm and 4°C for 5 min. Equal amounts of supernatant were diluted to a final concentration containing 60% methanol with LC−MS grade water. The samples were subsequently transferred to a fresh Eppendorf tube with a 0.22 μm filter and then centrifuged at 15000 × g at 4°C for 10 minutes. Finally, the filtrate was injected into the LC−MS/MS system for analysis.

### 2.6 UHPLC−MS/MS analysis

LC−MS/MS analyses were performed using a Vanquish UHPLC system (Thermo Fisher) coupled with an Orbitrap Q Exactive HF-X mass spectrometer (Thermo Fisher). Samples were injected onto a Hypersil Gold column (100 mm × 2.1 mm, 1.9 μm) using a 16-min linear gradient at a flow rate of 0.2 mL/min. The eluents for the positive polarity mode were eluent A (0.1% FA in water) and eluent B (methanol). The eluents for the negative polarity mode were eluent A (5 mM ammonium acetate, pH 9.0) and eluent B (methanol). The solvent gradient was set as follows: 2% B, 1.5 min; 2-100% B, 12.0 min; 100% B, 14.0 min; 100-2% B, 14.1 min; and 2% B, 16 min. The Q Exactive HF-X mass spectrometer was operated in positive/negative polarity mode with a spray voltage of 3.2 kV, a capillary temperature of 320°C, a sheath gas flow rate of 35 arb and an aux gas flow rate of 10 arb.

### 2.7 Metabolite identification

The raw data files generated by UHPLC−MS/MS were processed using Compound Discoverer 3.0 (CD 3.0, Thermo Fisher) to perform peak alignment, peak picking, and quantitation for each metabolite. The main parameters were set as follows: retention time tolerance, 0.2 min; actual mass tolerance, 5 ppm; signal intensity tolerance, 30%; signal/noise ratio, 3; and minimum intensity, 100000. Then, the peak intensities were normalized to the total spectral intensity. The normalized data were used to predict the molecular formula based on additive ions, molecular ion peaks and fragment ions. Then, peaks were matched with the mzCloud (https://www.mzcloud.org/) and ChemSpider (http://www.chemspider.com/) databases to obtain accurate qualitative and relative quantitative results. In addition, significantly different metabolites were selected, and their relative abundances were used as indicators for further analysis.

### 2.8 Statistical analysis

Significant differences were calculated by one-way ANOVA tests or T tests in SPSS 19 (IBM, USA). Different letters and asterisks (*) in the figure indicate statistical significance (*P<* 0.05). Unsupervised principal component analyses (PCA) and partial least squares discriminant analysis (PLS-DA) clustering methods were performed by MetaboAnalyst (http://www.metaboanalyst.ca/). Variable importance in the projection (VIP) is the weighted sum of squares of the PLS-DA analysis, which represents the contribution rate of metabolite differences in different groups. The fold change (FC) is the ratio of the mean of all replicate quantitative values for each metabolite in the comparison group. Combined with the T test, differential metabolites were screened by the following criteria: VIP >1.0, FC >1.2 or FC<0.833 and *P* value<0.05.

## 3 Results

### 3.1 Physiological characterization

Based on the previous experiment, the ripening of blueberry fruit can be divided into six stages (S1-S6) (**Figure 1A**). Then, after 20 and 25 days of spraying ABA treatment, the number of mature fruits (S6) was counted, and the proportion of mature fruits was calculated (**Figure 1B**). The results showed that 20 days after treatment, the fruit maturity rate (percentage of mature fruit) of the 1000 mg/L ABA treatment group was approximately 36%, which was significantly higher than that of the control group and 500 mg/L ABA treatment group (24%). On the 25th day after treatment, the fruit maturity rate of the 1000 mg/L ABA treatment group was approximately 53%, and that of the control group and 500 mg/L ABA treatment group was approximately 32% and 34%, respectively. The results showed that ABA treatment could promote blueberry fruit ripening.

In addition, we also found that under three treatments (control group, 500 mg/L and 1000 mg/L ABA treatment group), the hardness of blueberry fruits at six developmental stages gradually declined (**Figure 1C**). From stages S1 to S3, there was no significant difference in fruit hardness between the treatment group and the control group (the highest fruit hardness was 0.68 kg·cm^-2^ in the control group at the S1 stage). Notably, from the S4 stage, the fruit hardness of the treatment group and the control group began to decrease significantly, and the fruit hardness of the 1000 mg/L treatment group was significantly reduced by 14.29% and 9.52% compared to the control and 500 mg/L treatment groups at the S4 stage, respectively. In addition, fruit hardness at the S5 and S6 stages in the 1000 mg/L treatment group was significantly reduced by 13.88% and 8.65%, respectively, compared to the control group. According to the change in fruit hardness, it was speculated that the flesh tissue changed. Therefore, mature fruit slices at the S6 stage of the 0 and 1000 mg/L ABA treatment groups with significant differences in hardness were selected for microscopic observation by the semithin section method (**Figure 1D**). Through comparison, it was found that the flesh tissue of the 1000 mg/L ABA treatment group was obviously fragmented, while the flesh tissue of the control group was complete and full, with less fragmentation. The fragmentation of pulp tissue in the 1000 mg/L ABA treatment group may indicate higher fruit maturity, which is consistent with the hardness results.

### 3.2 Metabolite profiling

**Figure 2:**
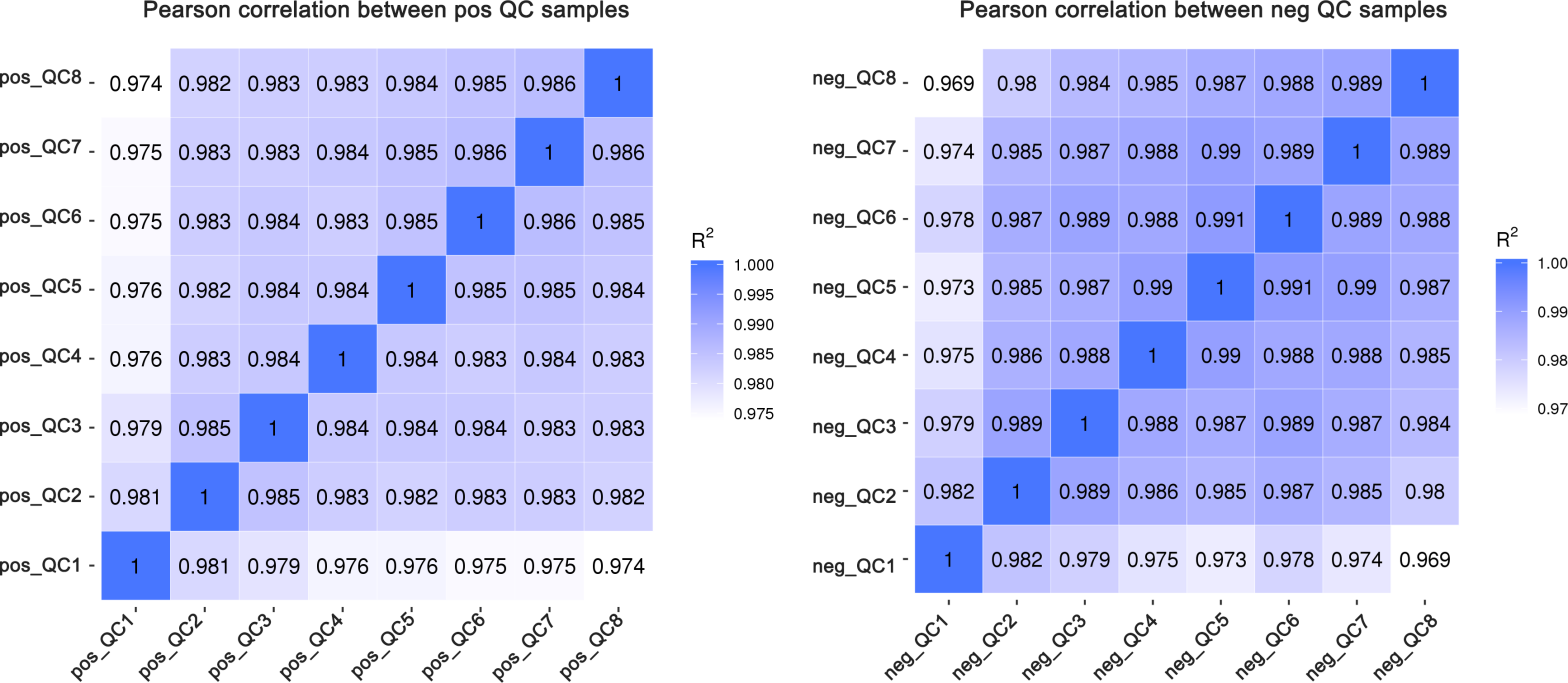
Pearson correlation between positive and negative quality control (QC) samples.

**Figure 3:**
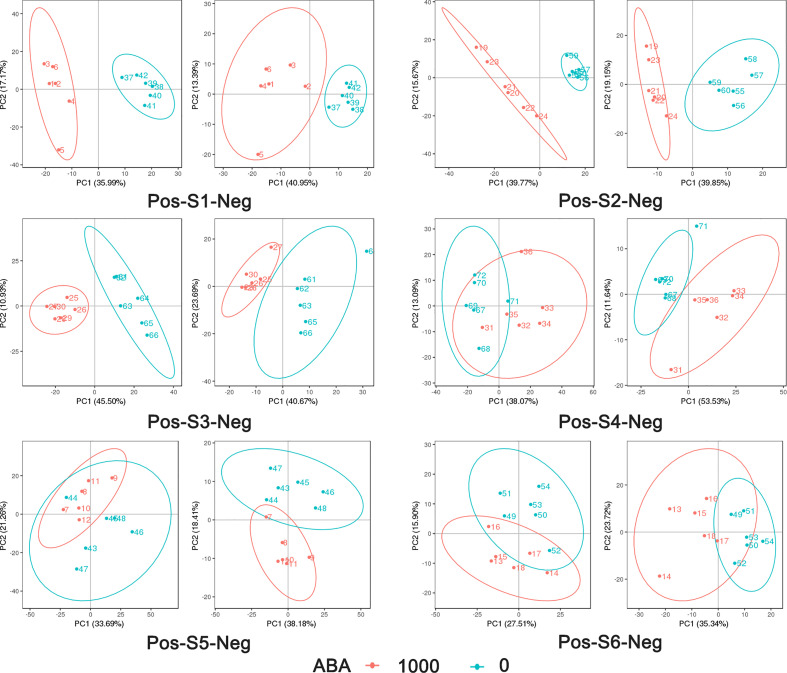
PCA analysis of metabolites in positive mode and negative mode in 6 stages: 1000 represents 1000 mg/L ABA treatment, and 0 represents 0 mg/L ABA treatment. The abscissa PC1 and ordinate PC2 represent the scores of the first and second principal components, respectively. The scatters of different colors represent the samples of different experimental groups, and the ellipse is the 95% confidence interval.

**Figure 4:**
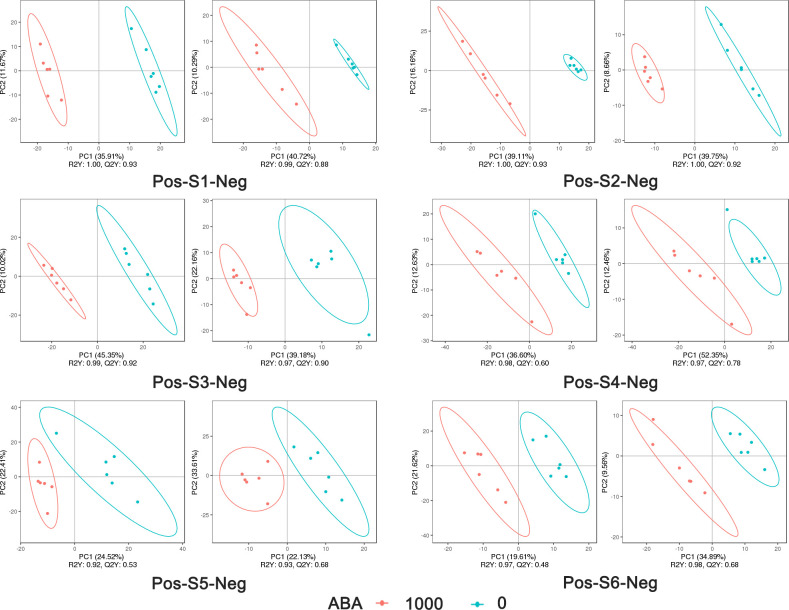
PLS-DA analysis of metabolites under the positive model and negative model in 6 stages: 1000 represents 1000 mg/L ABA treatment, and 0 represents 0 mg/L ABA treatment. The abscissa is the score of the sample on the first principal component; the ordinate is the score of the sample on the second principal component; R^2^Y represents the interpretation rate of the model, Q^2^Y is used to evaluate the predictive ability of the PLS-DA model, and when R^2^Y is greater than Q^2^Y, the model is well established.

**Figure 5:**
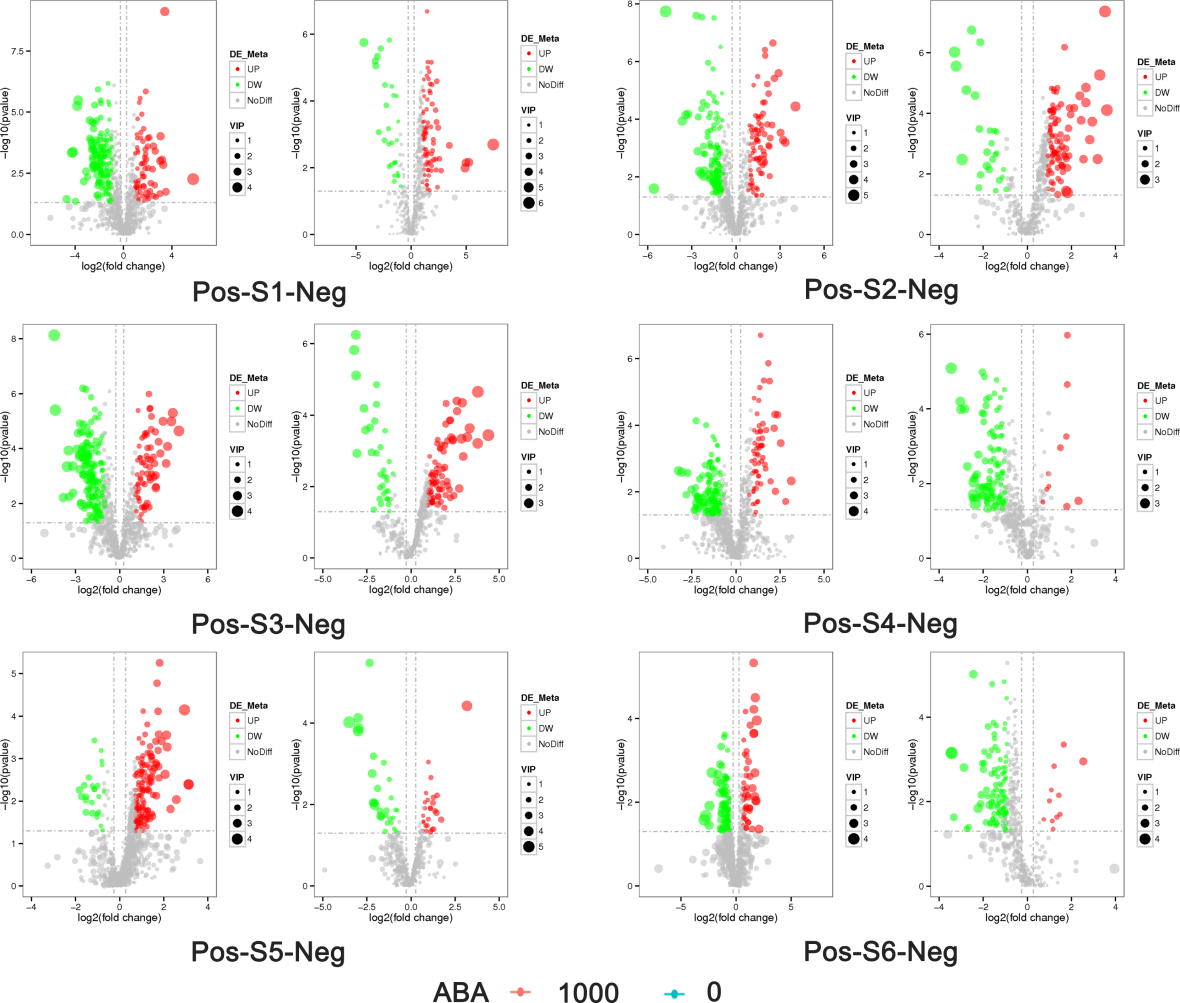
Volcano plot of the distribution of differential metabolites in blueberry fruits at the S1-S6 stages in the 0 and 1000 mg/L ABA treatment groups. Pos on the left side indicates positive ion mode, and neg on the right side indicates negative ion mode. The abscissa represents the log_2_ FC of metabolites in different groups, and the ordinate represents the level of significance. Each point in the volcano plot represents a metabolite, significantly upregulated metabolites are represented by red dots, significantly downregulated metabolites are represented by green dots, and the size of the dot represents the VIP value. UP, upregulation; DW, downregulation; NoDiff, no difference.

**Figure 6:**
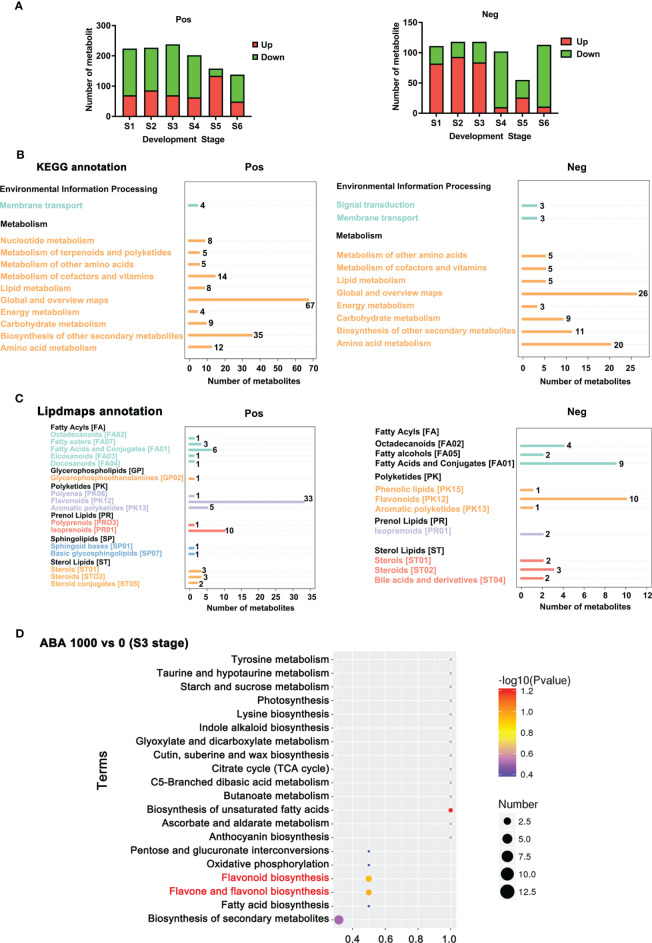
Annotated metabolites and total differential metabolites at 6 stages. **(A)** The number of differential metabolites (significant upregulation and downregulation) between 0 and 1000 mg/L ABA treatment at the S1-S6 period in positive ionization mode and negative ionization mode. **(B)** KEGG database annotation and enrichment of differential metabolites. The abscissa represents the number of metabolites, and the ordinate represents the annotated KEGG entries. **(C)** LIPID MAPS database annotation and enrichment of differential metabolites. The abscissa represents the number of metabolites, and the ordinate represents the annotated NMDB entries. **(D)** KEGG enrichment analysis of differential metabolites in blueberry fruits treated with 1000 mg/L vs. 0 mg/L. The abscissa is the name of the metabolic pathway, and the ordinate is the number of differential metabolites in the corresponding metabolic pathway, or the number of total metabolites identified in that pathway, with larger values indicating higher enrichment of differential metabolites in that pathway. The color of the dot represents the p-value of the hypergeometric test, and the smaller the value, the more reliable and statistically significant the test is. The size of the dots represents the number of differential metabolites in the corresponding pathway; the larger the value, the more differential metabolites are present in the pathway.

The 0 and 1000 mg/L ABA treatments were selected for nonbiased global metabolomics (nontarget metabolomics) based on UHPLC−MS/MS. Quality control (QC) analysis showed that the quality of metabolomics was stable and adequate (**Figure 2**). A total of 1145 metabolites and 575 metabolites were detected and annotated in positive mode (**Table S1**) and negative mode (**Table S2**), respectively. Principal component analysis (PCA) (**Figure 3**) and partial least squares discrimination analysis (PLS-DA) (**Figure 4**) were performed to systematically profile metabolic variances between the two groups. Score plots of samples are well separated in S1, S2 and S3, suggesting a clear distinction in metabolite accumulation. In contrast, the scores plotted in S4, S5 and S6 intersect to different degrees. A volcano map can directly show the overall distribution of differential metabolites (**Figure 5**). Compared with the S1-S3 stages, the number of different metabolites in blueberry fruits during the S4-S6 stages was significantly reduced. The total amount of upregulated and downregulated differential metabolites of blueberry fruit S1-S6 in the 0 and 1000 mg/L ABA treatment groups at each stage showed that the total amount of differential metabolites increased continuously at the S1-S3 stages and peaked at the S3 stage (**Figure 6A**). The upregulation and downregulation of differential metabolites detected in the two modes were irregular. Differential metabolites were annotated by Kyoto Encyclopedia of Genes and Genomes (KEGG) (**Figure 6B**) and Lipid metabolites and pathways strategy (LIPID MAPS) (**Figure 6C**). In the LIPID MAPS annotation, there were 33 metabolites in positive mode and 10 metabolites in negative mode involved in flavonoid metabolism. During the S3 period, when the most differential metabolites were found, the flavonoid biosynthesis pathway and flavone and flavonol biosynthesis pathway were significantly enriched in blueberry fruits under positive mode (**Figure 6D**). In addition, the top 10 upregulated (**Table S3**) and downregulated (**Table S4**) metabolites were separated. Two compounds were noteworthy: gallic acid was strikingly upregulated from S1-S3, and chlorogenic acid was significantly and continuously downregulated from S1 to S6.

### 3.3 ABA concentration and stress resistance

**Figure 7:**
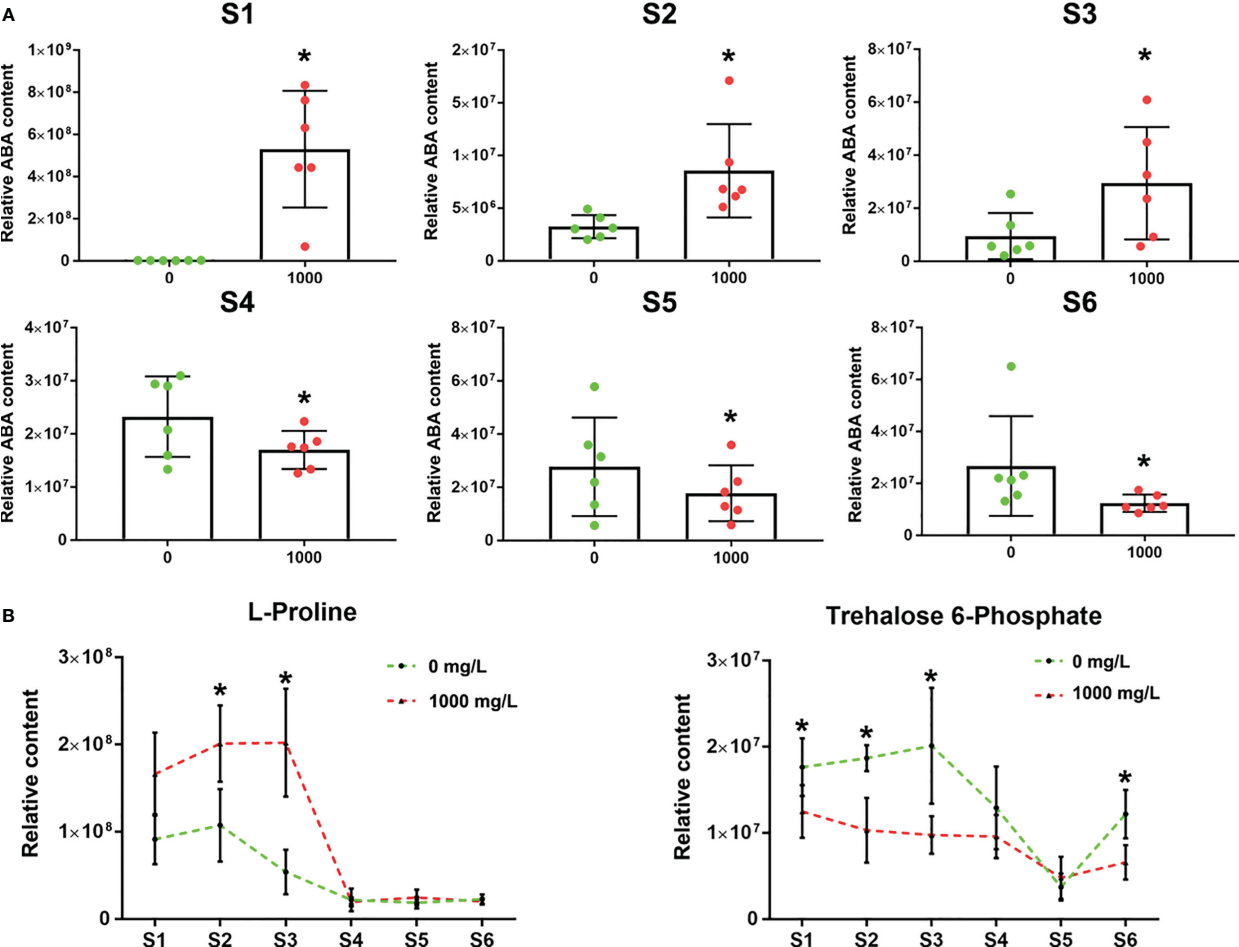
Changes in relative ABA content and resistance to adversity. **(A)** Relative ABA content in the 0 and 1000 mg/L ABA treatment groups in 6 stages. **(B)** Relative L-proline and trehalose-6-phosphate contents under 0 and 1000 mg/L ABA treatment from S1 to S6. An asterisk (*) indicates a significant difference between different treatments during the same period according to Tukey’s test (*P*< 0.05).

The ABA relative content in the control group continued to increase from S1 to S4 but began to decrease from S4 to S5 and then increased from S5 to S6 (**Figure 7A**). The mean ABA relative content in the ABA group was higher than that in the control group at the S1-S3 stage but lower than that in the control group at the 4-6 stage (**Figure 7A**). From stages S1-S3, the L-proline relative content in the 1000 mg/L group was significantly higher than that in the control group, and the trehalose-6-phosphate relative content in the 1000 mg/L group was significantly lower than that in the control group (**Figure 7B**). The change in metabolite levels of the two stress resistance markers was consistent with that of endogenous ABA. During the S4-S6 period, the two stress-resistance metabolites of the treatment group and the control group tended to be the same, showing that the effect of exogenous ABA was reduced.

### 3.4 Flavonoid pathway

**Figure 8:**
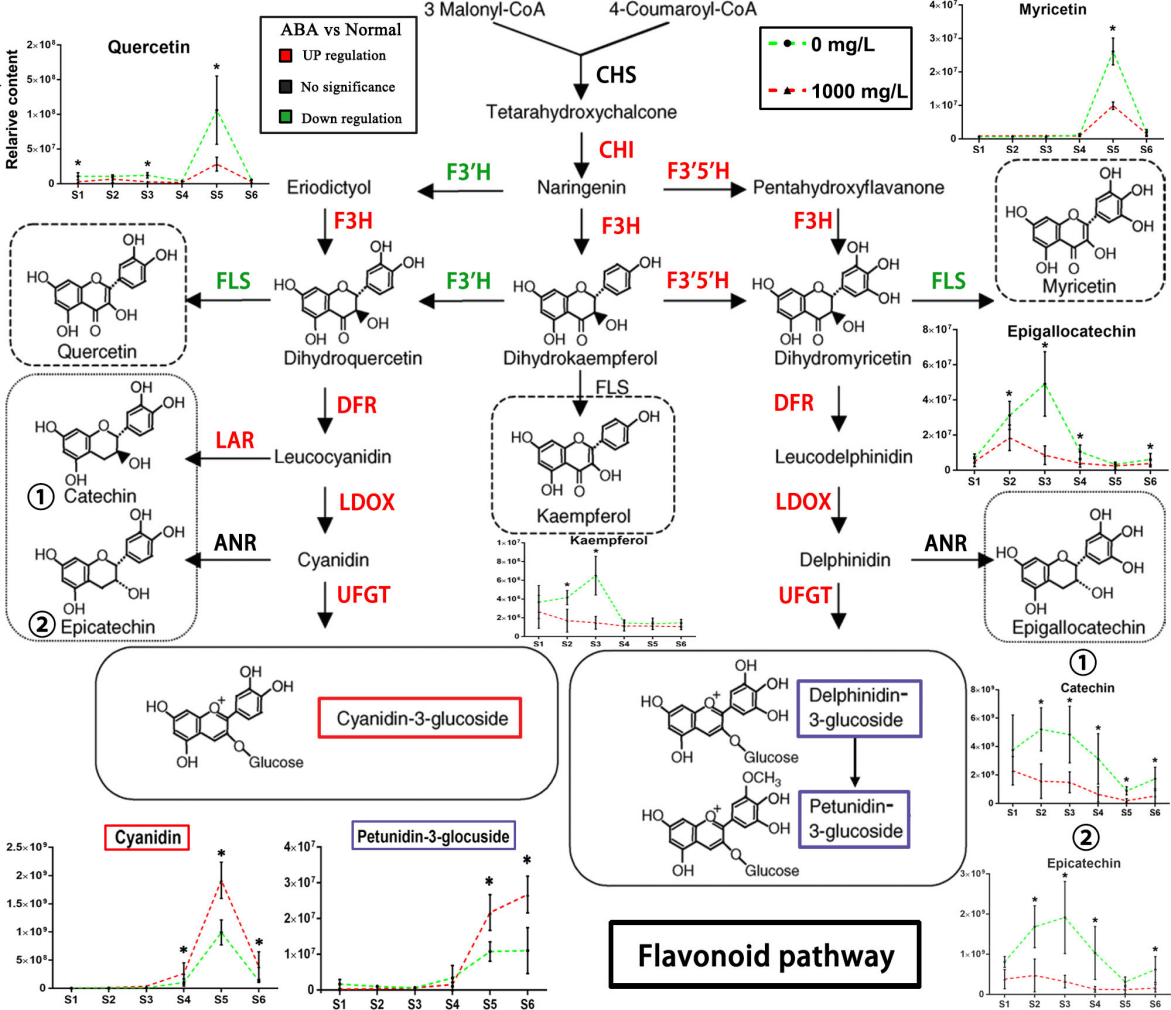
Schematic diagram of the flavonoid pathway. The changes in key metabolites in blueberry fruits of the flavonoid pathway during S1-S6 in the 0 and 1000 mg/L ABA treatment groups are illustrated. The metabolites of key nodes are circled with dashed lines, and the corresponding broken line graph shows the changes in relative contents in the S1-S6 period after treatment. CHS, chalcone synthase; CHI, chalcone isomerase; F3H, flavanone 3-hydroxylase; F3’H, flavonoid 3’-hydroxylase; F3’5’H, flavonoid 3’,5’-hydroxylase; DFR, dihydroflavonol 4-reductase; LAR, leucoanthocyanidin reductase; FLS, flavonol synthase; ANR, anthocyanidin reductase; LDOX, leucocyanidin oxygenase; UFGT, UDP-glucose: flavonoid-3-*O*-glycosyltransferase. An asterisk (*) indicates a significant difference between different treatments during the same period according to Tukey’s test (*P*< 0.05).

For the flavonoid pathway, the 1000 mg/L group differed from the control group at the important metabolite level, and the differences were found and annotated by KEGG (**Figure 8**). Cyanindin is a precursor of red anthocyanins. Its content is consistent with the coloration change, and its content increased more than that of the control group from S3. Similarly, the level of blue anthocyanin petunidin-3-glucoside increased more in the treatment group than in the control group when the blueberry fruits turned purple. Quercetin, catechin and epicatechin are downstream metabolites of flavonoid 3’-hydroxylase (F3’H), showing a persistently lower content from S1 to S6. Myricetin and epigallocatechin are downstream metabolites of flavonoid flavonoid-3’,5’-hydroxylase (F3’5’H) and were present at lower levels than in the control group in S5 and S3, respectively.

### 3.5 Bioactive metabolite levels and antioxidant capacity

**Figure 9:**
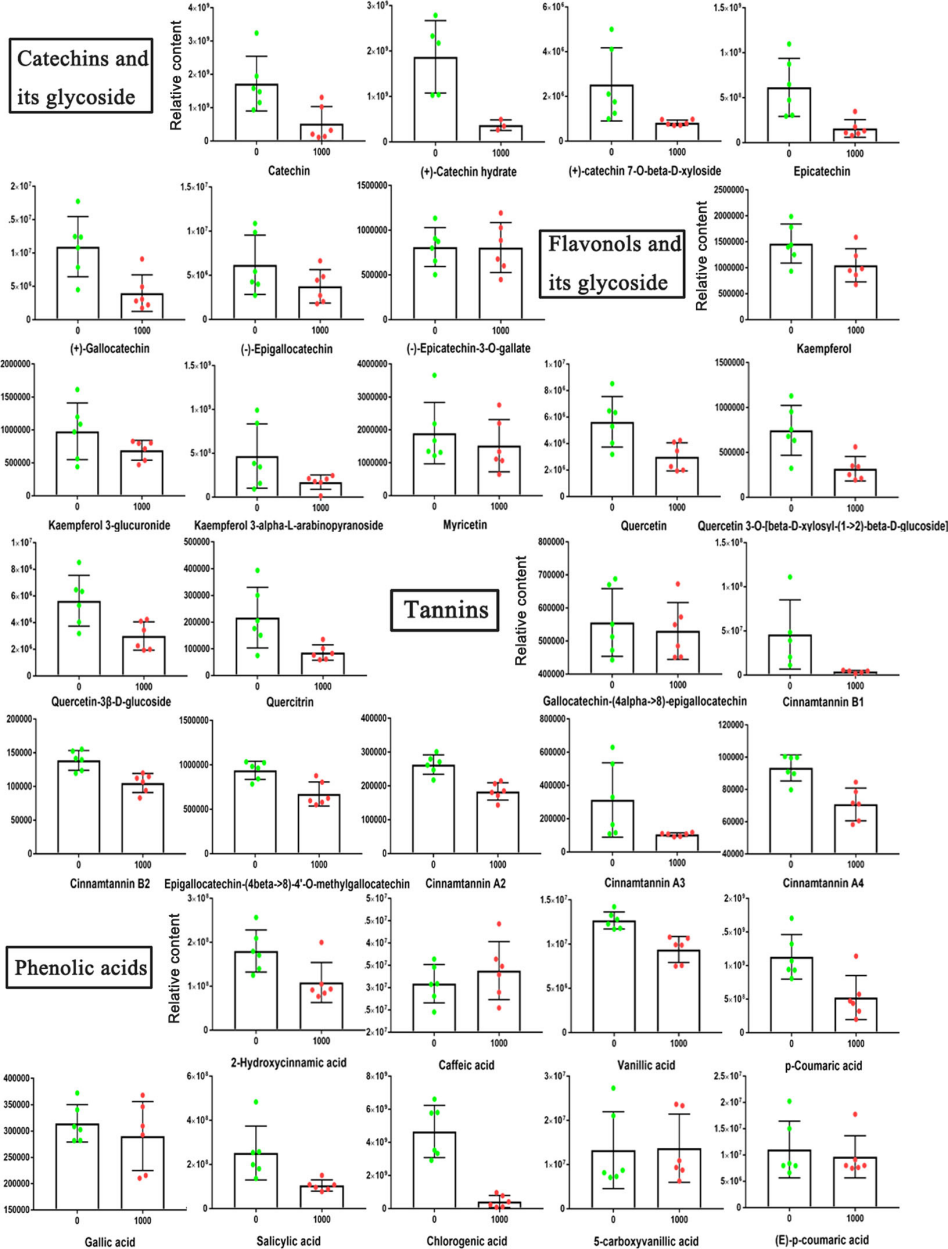
Relative abundances of bioactive metabolites under 0 and 1000 mg/L ABA treatment. An asterisk (*) indicates a significant difference between different treatments during the same period according to Tukey’s test (*P*< 0.05).

Due to the significant changes in metabolites in the flavonoid biosynthesis pathway, we further analyzed the changes in other flavonoid metabolites in blueberry fruits treated with ABA. The representative metabolites among catechins, flavonols, tannins and phenolic acids in mature fruits are shown (**Figure 9**). Overall, most of their contents were reduced to some extent under the 1000 mg/L ABA treatment. The two metabolites were noteworthy: gallic acid was higher at the S1-S3 stages and returned to normal levels in the ripe fruit, but chlorogenic acid remained at low levels in the ripe fruit. Because of the changes in bioactive metabolites, we further studied the antioxidant capability of fruits. The reducing ability of the 1000 mg/L ABA treatment was higher than that of the 0 mg/L treatment (**Figure S1A**). In addition, the DPPH radical scavenging activity assay (**Figure S1B**) also showed the same result.

### 3.6 Changes in anthocyanin components

According to previous research by our research group, the total anthocyanins of fruits can be detected from the S4 stage, and the total anthocyanin content increases with the development of fruits (Han et al., 2022). Based on this, the anthocyanin components in blueberry fruits of the 0 (CK) and 1000 mg/L ABA treatment groups with significant differences in anthocyanin content were analyzed by LC−MS. In each of the S3-S6 stages, five pigments, petunidin, cyanidin, delphinidin, pelargonidin and peonidin, were detected in blueberry fruits, and the content of cyanidin and pelargonidin in the fruits at the S3 stage of the 1000 mg/L ABA treatment group was significantly increased by 10.10% and 15.43%, respectively, compared to the control group. Meanwhile, the content of each pigment in the fruits of the 1000 mg/L ABA treatment group under the S4-S6 stage was significantly higher than that of the control fruits in the same period, except for cyanidin and pelargonidin. Notably, the content of cyanidin and delphinidin was higher at the beginning of blueberry fruit development (S3), with a decreasing trend at the later stage, while their content showed a certain increase after fruit ripening. In addition, except for cyanidin and pelargonidin, the contents of which decreased sharply from S3-S5, the other pigments all showed a steady increasing trend during fruit development and maturity and reached the highest content when the fruit was fully mature at S6 (**Table 1**).

**Table 1:** Effect of ABA treatment on the type and content of anthocyanins in blueberry fruit.

Sample	Petunidin (ng/g)	Cyanidin (ng/g)	Delphinidin (ng/g)	Pelargonidin (ng/g)	Peonidin (ng/g)
CK-S3	48.23 ± 5.62	91758.27 ± 625.57	123.28 ± 7.67	1796.51 ± 82.64	146.97 ± 4.88
CK-S4	299.32 ± 13.12	46504.10 ± 95.89	152.73 ± 9.72	754.10 ± 9.58	523.28 ± 7.67
CK-S5	3448.22 ± 79.37	40904.78 ± 841.87	1669.54 ± 34.29	738.30 ± 73.49	2493.31 ± 84.85
CK-S6	105710.46 ± 401.98	54684.30 ± 43.99	57742.86 ± 56.36	737.21 ± 75.98	26958.68 ± 142.68
ABA-S3	24.82 ± 1.43	101021.94 ± 244.63 *	89.92 ± 8.05	2073.74 ± 10.07 *	159.35 ± 25.17
ABA-S4	965.49 ± 63.68 *	45510.59 ± 322.03	412.22 ± 76.02 *	795.70 ± 21.79	1185.53 ± 26.87 *
ABA-S5	6940.35 ± 87.71 *	34488.30 ± 409.35	3102.04 ± 67.83 *	544.73 ± 6.84	2714.03 ± 50.87 *
ABA-S6	118278.30 ± 533.23 *	74511.32 ± 213.29 *	61250.18 ± 26.15 *	1128.56 ± 99.34 *	33241.05 ± 186.26 *

An asterisk (*) indicates a significant difference between different treatments during the same period according to Tukey’s test (P< 0.05).

## 4 Discussion

As a nutritious fruit, blueberry is known to be rich in antioxidant anthocyanins and bioactive metabolites (Borges et al., 2010; Nile and Park, 2014). The aim of this study was to find and illustrate a conventional method to increase the anthocyanin content of blueberries. Previous studies on non-climacteric fruits, such as grape (Sandhu et al., 2011; Yamamoto et al., 2015) and strawberry (Li et al., 2014), found that exogenous ABA on veraison (the beginning of fruit maturity) could increase the total anthocyanin content, such as that in grape (Sandhu et al., 2011; Yamamoto et al., 2015) and strawberry (Li et al., 2014). However, these studies only involved changes in anthocyanin content and did not study changes in the overall metabolite level. As a non-climacteric fruit, blueberry begins to accumulate anthocyanins from the late green fruit stage (Zifkin et al., 2012). Two studies on the external application of ABA in blueberry also did not go deep into the overall metabolite change but were limited to the change in anthocyanin content (Buran et al., 2012; Oh et al., 2018). There are many methods in production and cultivation practice that can increase the endogenous ABA content of fruits and anthocyanin content by artificially increasing the stress conditions. Therefore, it is of great practical significance to study the effects of exogenous ABA treatment on fruit development and secondary metabolites. To date, there are few studies on the effect of the external application of ABA on the change in the overall metabolites of non-climacteric fruits. Based on these findings, physiological experiments and nontarget metabolomics were combined to systematically and comprehensively demonstrate the effects of high concentrations of ABA on mature green blueberry fruits at the metabolite level.

Exogenous ABA accelerated fruit ripening in this study, which was consistent with the previous results of other non-climacteric fruits (Li et al., 2014; Yamamoto et al., 2015). In addition, we found that the endogenous ABA relative content and stress resistance of blueberry fruits increased from S1 to S3 (approximately 15-20 days) (**Figure 7A**). Proline is a crucial osmotic adjustment in plants and accumulates when plants are under environmental stress (Szabados and Savouré, 2010). Trehalose-6-phosphate serves as a signaling molecule that is negatively correlated with the response of plants to conditions that result in starvation (O'Hara et al., 2013). In these stages, the proline content increased, and the trehalose-6-phosphate content decreased, indicating that the fruit had relatively strong resistance to adversity (**Figure 7B**). The anthocyanin content is most important for blueberry fruit, and the higher the anthocyanin content is, the greater the reducing ability of blueberry fruits (Han et al., 2022). Meanwhile, the results of this study also showed that the reducing and DPPH assays of blueberry fruits under 1000 mg/LABA treatment increased by 30.95% and 50.98%, respectively, compared to the control group (**Figure S1**). Thus, the reducing and DPPH assays also support the result that blueberry fruits under 1000 mg/L ABA treatment have a stronger reducing ability.

Berli et al. (2011) treated ‘Malbec’ cultivar grapes 45 days after flowering with 1 mM (264 mg/L) ABA. The results showed that the contents of delphinidin, cyanidin, and petunidin in the mature fruits 126 days after flowering were significantly higher than those in the control group. In the experimental results of Sandhu et al. (2011), 300 ppm ABA was applied to ‘Noble’ and ‘Alachua’ grapes to increase the content of total anthocyanins and phenols in the fruit, as well as the total reducibility of the fruit. Yamamoto et al. (2015) found that the application of ABA to ‘Isabel’ grapes during the color change period would accelerate the color change in fruits and increase the anthocyanin content in mature fruits. For strawberry, Jia et al. (2011) found that 0.5 µM ABA treatment could accelerate fruit ripening and increase the anthocyanin content of mature fruit in the color conversion period. In this study, we found that the contents of three anthocyanins, petunidin, delphinidin, and peonidin, increased rapidly with the development and maturity of the fruit, while the contents of cyanidin and pelargonidin showed a gradual rebound change. Furthermore, the contents of the five anthocyanins in the blueberry fruits treated with 1000 mg/L ABA were significantly higher than those in the control group at S4-S6 stages, except for the contents of cyanidin and pelargonidin at the S4-S5 stages (**Table 1**). In summary, ABA treatment can accelerate fruit ripening, increase anthocyanin accumulation and improve antioxidant capacity, similar to the above anthocyanin-related studies.

In this study, we found that flavonals temporarily decreased in content during maturation, and the final average value was lower than that of the treatment group under 1000 mg/L ABA treatment, such as myricetin, quercetin and kaempferol. This result conflicts with previous research on grapes (Sandhu et al., 2011), which showed that the flavonol content is higher in mature fruits. However, the RNA-seq data of exogenous ABA on grapes at 22 h and 44 h showed that chalcone synthase transcripts were downregulated at 22 h and returned to normal expression at 44 h (Pilati et al., 2017). Considering the change in the first enzyme in the flavonoid pathway and our nontargeted metabolomics results (**Figure 4**), we speculate that there is a competitive relationship between flavonol and anthocyanin content, so there is a trade-off phenomenon.

## 5 Conclusions

The application of 1000 mg/L ABA in the late green fruit stage (S1) of blueberry significantly affected the changes in the overall level of fruit metabolites during the S1-S6 period. The number of differential metabolites was the largest in the S3 period. Forty-six differential metabolites were annotated in the KEGG database, mainly related to the synthesis pathway of secondary metabolites. LIPID MAPS notes 43 differential metabolites, which are mainly related to the flavonoid biosynthesis pathway. In summary, exogenous ABA treatment can change the endogenous ABA level, accelerate the ripening of blueberry fruit, increase the accumulation of anthocyanin components in fruit and improve its antioxidant capacity.

## Data availability statement

The original contributions presented in the study are included in the article/**Supplementary Material**. Further inquiries can be directed to the corresponding authors.

## Author contributions

HY, YW, and TH: Writing – original draft and Investigation. LL: Resources. WL: Writing– review and editing. WW: Writing– review and editing. All authors contributed to the article and approved the submitted version.

## Supplementary material

Supplementary Figure 1 Reducing ability of fruits under 0 and 1000 mg/L ABA treatment. Ascorbic acid was used as a positive control. **(A)** The reducibility of fruits. **(B)** Scavenging rate of DDPH. An asterisk (*) indicates a significant difference between different treatments during the same period according to Tukey’s test (*P*< 0.05).

Supplementary Table 1 A total of 1145 metabolites were detected and annotated in blueberry fruits at ABA treatment 1000 mg/L vs. 0 mg/L (the control) in positive mode.

Supplementary Table 2 A total of 575 metabolites were detected and annotated in blueberry fruits at ABA treatment 1000 mg/L vs. 0 mg/L (the control) in negative mode.
