# Supplementary figures and images for: Quality analysis and metabolomic profiling of the effects of exogenous abscisic acid on rabbiteye blueberry
Source: Front Plant Sci. 2023 Jul 10;14:1224245. doi: 10.3389/fpls.2023.1224245 (PMC10364122; doi:10.3389/fpls.2023.1224245)

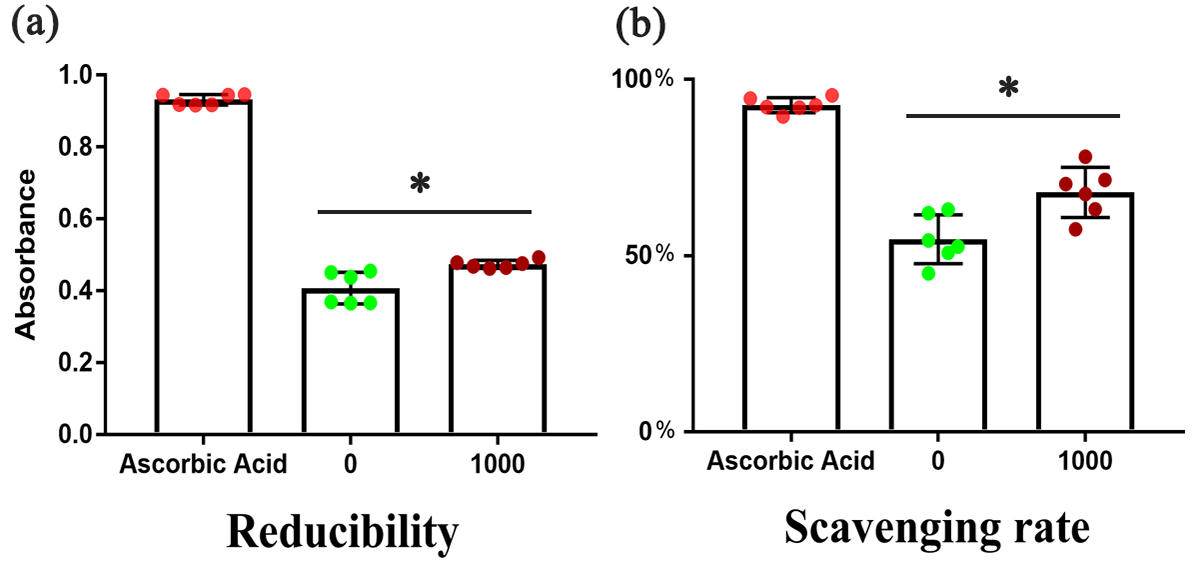

Supplement: Supplementary Figure 1 — Reducing ability of fruits under 0 and 1000 mg/L ABA treatment. Ascorbic acid was used as a positive control. (A) The reducibility of fruits. (B) Scavenging rate of DDPH. An asterisk (*) indicates a significant difference between different treatments during the same period according to Tukey’s test (P< 0.05). [file Image_1.tif]
