# Supplementary material for: Quality analysis and metabolomic profiling of the effects of exogenous abscisic acid on rabbiteye blueberry
Source: Front Plant Sci. 2023 Jul 10;14:1224245. doi: 10.3389/fpls.2023.1224245 (PMC10364122; doi:10.3389/fpls.2023.1224245)
Supplement: Supplementary file 4 [file Table_3.docx]

**Table S3.** Top 10 upregulated metabolites in 6 growth stages in blueberry fruits at ABA treatment 1000 mg/L vs. 0 mg/L (the control).

| Stage 1 | log2FC | Stage 2 | log2FC | Stage 3 | log2FC |
| --- | --- | --- | --- | --- | --- |
| (+)-Abscisic acid | 5.75 | MFCD19443524 | 4.05 | 3-Hydroxyvaline | 4.38 |
| 1-(4-Pyridinyl)-4-piperidinecarboxamide | 5.24 | Gallic acid | 3.97 | 7-(Methylsulfanyl)[1,3]thiazolo[4,5-g][1,3]benzothiazol-2-amine | 4.03 |
| Jasmonal | 4.98 | 1-(4,6-Dimethoxy-1-benzofuran-2-yl)ethanone | 3.61 | Gallic acid | 3.90 |
| streptidine | 4.90 | 9-Oxo-9H-fluorene-2,7-disulfonyl dichloride | 3.36 | FMNH2 | 3.79 |
| 3-Hydroxyvaline | 3.97 | FMNH2 | 3.52 | 1-(4,6-Dimethoxy-1-benzofuran-2-yl)ethanone | 3.78 |
| 7-(Methylsulfanyl)[1,3]thiazolo[4,5-g][1,3]benzothiazol-2-amine | 3.51 | Diisodecyl phthalate | 3.29 | Quillaic Acid | 3.77 |
| Dehydrovomifoliol | 3.47 | (24S)-ergostan-3b,5a,6b,18,25-pentaol 18,25-diacetate | 3.28 | 9-Oxo-9H-fluorene-2,7-disulfonyl dichloride | 3.61 |
| Amide C18 | 3.41 | 2,4-Dinitro-3'-nitrodiphenyl ether | 3.21 | (3beta)-3-(Nonyloxy)olean-12-ene | 3.52 |
| Gallic acid | 3.40 | taxifolin | 3.19 | Hexose | 3.47 |
| 2-(Difluoromethyl)-9,9-dimethyl-9H-fluorene | 3.29 | okenone | 3.05 | Diisodecyl phthalate | 3.32 |
| Stage 4 | log2FC | Stage 5 | log2FC | Stage 6 | log2FC |
| marrubin | 3.16 | Ethyl 2-(2-naphthyl)-2-[(trifluoromethyl)sulfanyl]-4-pentenoate | 3.65 | Quizalofop-P-tefuryl | 3.95 |
| (+)-Staurosporine | 3.13 | Mitobronitol | 3.17 | Cyclododecyne | 2.90 |
| Bruceine A | 3.04 | 7-Isoquinolinyl trifluoromethanesulfonate | 3.13 | 7-Isoquinolinyl trifluoromethanesulfonate | 2.63 |
| Boldione | 3.02 | (+)-Staurosporine | 3.12 | Mitobronitol | 2.54 |
| 2-Chloro-6-(hydroxyamino)phenol | 2.89 | 1-(3,5-Dichloro-2,6-dihydroxy-4-methoxyphenyl)-1-hexanone | 3.08 | N-(3,4-Dimethylphenyl)-9,10-dioxo-9,10-dihydro-1-anthracenesulfonamide | 2.30 |
| 3,3-Bis(1,3-thiazol-2-ylamino)-1,2-propanediol | 2.82 | nimidane | 2.94 | DL-Carbidopa | 2.30 |
| 4-(Dodecyloxy)-2,2'-bis(ethoxymethoxy)-4'-ethynylbiphenyl | 2.77 | MFCD15146035 | 2.89 | Etoglucid | 2.22 |
| MFCD19443524 | 2.54 | 4-Aminobenzoate | 2.60 | Diosmin | 2.20 |
| bengazole A | 2.52 | hymecromone | 2.60 | (+)-Staurosporine | 2.09 |
| Quillaic Acid | 2.37 | Gemfibrozil | 2.57 | Tosufloxacin | 1.91 |

Note: Log 2FC indicates the degree of metabolite difference between 1000 mg/mL ABA treatment and 0 mg/L (the control).
