# Supplementary material for: Quality analysis and metabolomic profiling of the effects of exogenous abscisic acid on rabbiteye blueberry
Source: Front Plant Sci. 2023 Jul 10;14:1224245. doi: 10.3389/fpls.2023.1224245 (PMC10364122; doi:10.3389/fpls.2023.1224245)
Supplement: Supplementary file 5 [file Table_4.docx]

**Table S4.** Top 10 downregulated metabolites in 6 growth stages in blueberry fruits at ABA treatment 1000 mg/L vs. 0 mg/L (the control).

| Stage 1 | log2FC | Stage 2 | log2FC | Stage 3 | log2FC |
| --- | --- | --- | --- | --- | --- |
| 3-hydroxybenzylhydrazine | -6.03 | **2-Chloro-6-(hydroxyamino)phenol** | -5.59 | **2-Chloro-6-(hydroxyamino)phenol** | -5.12 |
| 5,5'-Dehydrodivanillate | -4.69 | 7-Hydroxy-6-[(2-oxo-2H-chromen-7-yl)oxy]-2H-chromen-2-one | -4.79 | 7-Hydroxy-6-[(2-oxo-2H-chromen-7-yl)oxy]-2H-chromen-2-one | -4.46 |
| **2-Chloro-6-(hydroxyamino)phenol** | -4.48 | 5,5'-Dehydrodivanillate | -4.42 | Cinnamtannin A3 | -4.38 |
| aldioxa | -4.29 | Cinnamtannin A3 | -3.66 | Aripiprazole | -3.83 |
| Cinnamtannin B1 | -4.25 | DL-Carbidopa | -3.45 | Tetrakis(4-nitrosophenyl)silane | -3.58 |
| Combretastatin A-1 | -4.17 | p-Chloroacetophenone | -3.42 | Cinnamtannin B1 | -3.48 |
| hexanoyl-CoA | -4.16 | 1-(1H-Indol-3-ylcarbonyl)-9H-beta-carboline-3-carboxylic acid | -3.30 | fijianolide D | -3.46 |
| all-trans-retinal | -4.16 | Bromoxanide | -3.27 | Mahuannin D | -3.44 |
| (+)-Fluprostenol | -3.96 | **Chlorogenic acid** | -3.23 | **Chlorogenic acid** | -3.22 |
| p-Chloroacetophenone | -3.82 | aldioxa | -2.96 | Benzyl beta-D-xylopyranoside | -3.21 |
| Stage 4 | log2FC | Stage 5 | log2FC | Stage 6 | log2FC |
| 3-Phenyl-4-(phenylsulfanyl)butanal | -4.12 | (1S)-1,5-Anhydro-1-benzyl-D-galactitol | -4.90 | Guanine | -7.02 |
| TI1940000 | -3.45 | tectorigenin | -3.51 | Benzyl beta-D-xylopyranoside | -3.91 |
| Cinnamtannin B1 | -3.26 | Propicillin | -3.27 | 2-amino-2,3,7-trideoxy-D-lyxo-hept-6-ulosonic acid | -3.61 |
| Melibiose | -3.07 | 1-(1H-Indol-3-ylcarbonyl)-9H-beta-carboline-3-carboxylic acid | -3.00 | 1-(1H-Indol-3-ylcarbonyl)-9H-beta-carboline-3-carboxylic acid | -3.44 |
| hexanoyl-CoA | -3.07 | Benfotiamine | -3.00 | **Chlorogenic acid** | -3.43 |
| **Chlorogenic acid** | -3.04 | **Chlorogenic acid** | -2.97 | Flufenamic Acid | -3.33 |
| Epicatechin | -3.02 | Gallic acid | -2.82 | Silafluofen | -2.95 |
| 1-(1H-Indol-3-ylcarbonyl)-9H-beta-carboline-3-carboxylic acid | -2.85 | guajavarin | -2.35 | Fludiazepam | -2.92 |
| Cinnamtannin A3 | -2.82 | 2-{[(3-Hydroxy-2-oxo-2,3-dihydro-1H-indol-3-yl)acetyl]amino}succinate | -2.20 | Cinnamtannin B1 | -2.89 |
| 5-Methoxy-3-indoleaceate | -2.78 | Catechin | -2.20 | TI1940000 | -2.88 |

Note: Log 2FC indicates the degree of metabolite difference between 1000 mg/mL ABA treatment and 0 mg/L (the control).
